# Utility of the JAX Clinical Knowledgebase in capture and assessment of complex genomic cancer data

**DOI:** 10.1038/s41698-018-0073-y

**Published:** 2019-01-15

**Authors:** Sara E. Patterson, Cara M. Statz, Taofei Yin, Susan M. Mockus

**Affiliations:** The Jackson Laboratory for Genomic Medicine, Farmington, CT USA

## Abstract

Cancer genomic data is continually growing in complexity, necessitating improved methods for data capture and analysis. Tumors often contain multiple therapeutically relevant alterations, and co-occurring alterations may have a different influence on therapeutic response compared to if those alterations were present alone. One clinically important example of this is the existence of a resistance conferring alteration in combination with a therapeutic sensitizing mutation. The JAX Clinical Knowledgebase (JAX-CKB) (https://ckb.jax.org/) has incorporated the concept of the complex molecular profile, which enables association of therapeutic efficacy data with multiple genomic alterations simultaneously. This provides a mechanism for rapid and accurate assessment of complex cancer-related data, potentially aiding in streamlined clinical decision making. Using the JAX-CKB, we demonstrate the utility of associating data with complex profiles comprising ALK fusions with another variant, which have differing impacts on sensitivity to various ALK inhibitors depending on context.

## Database: Online resource captures complex genomics to guide drug decisions

An online repository of genomic and clinical data offers a powerful tool for oncologists to tailor therapeutic decision-making to the complex molecular landscape of a patient’s tumor. Susan Mockus and colleagues from the Jackson Laboratory for Genomic Medicine in Farmington, Connecticut, USA, describe the concept of a ‘complex molecular profile’ included in the JAX Clinical Knowledgebase of curated data on genomic determinants of response to anti-cancer agents. This concept allows researchers to tie therapeutic outcomes data to many different genomic alterations at once. As a proof of concept, the researchers used the knowledgebase to look at tumor sensitivity to ALK inhibitors — drugs that block common gene fusions involving *ALK*. They showed that the impact of resistance-conferring mutations depended on the nature of *ALK*’s fusion partner. The findings highlight the importance of considering all relevant genomic changes when choosing a course of therapy.

## Introduction

Tumors are genomically complex, often arising as the result of a combination of alterations that ultimately lead to uncontrolled cellular proliferation. Once formed, tumors continue to evolve, acquiring additional alterations that contribute to temporal and spatial tumor heterogeneity and which potentially enable the tumor to evade immune response or therapeutic intervention.^1–3^ Historically, precision oncology has been approached through targeting a single driver alteration, potentially in the presence of other relevant mutations. As knowledge of tumor biology continues to grow, it becomes increasingly apparent that alterations often work in concert to affect pathway regulation and therapeutic efficacy highlighting the need for new targeted therapies and combination therapies that target multiple alterations simultaneously.^2,4–7^ For example, in lung cancer, the notion of the single driver alteration has been dispelled by studies demonstrating multiple co-occurring driver alterations in patients with EGFR-mutant non-small cell lung cancer, including mutations in PIK3CA or CTNNB1.^8–10^

One important aspect of tumor biology is the acquisition of mutations that confer therapeutic resistance, which often arise in the context of a therapy sensitizing mutation, and frequently occur following intervention with targeted therapies.^4,11^ One well-known example of this is the EGFR T790M mutation, which commonly occurs in lung cancer patients with EGFR-inhibitor sensitizing mutations following treatment with EGFR tyrosine kinase inhibitors and renders first and second-generation inhibitors ineffective.^8,12–14^ Resistance to EGFR inhibitors in the context of an EGFR activating mutation can also occur through the acquisition of a number of other types of alterations, such as MET amplification or a CCDC6-RET fusion.^15–17^ Secondary mutations can also confer therapeutic resistance via other mechanisms, such as resistance to PARP inhibitors in patients with BRCA1 frameshift mutations via acquisition of mutations that restore the BRCA1 open reading frame, termed reversion mutations.^18^ Knowledge surrounding the varied types of resistance mechanisms that can arise, as well as ways to circumvent therapeutic resistance, is key to effective patient treatment.

Therapeutic resistance is exemplified by the acquisition of specific alterations in the context of ALK fusions following treatment with ALK inhibitors. ALK is a receptor protein tyrosine kinase that belongs to the insulin receptor superfamily of protein tyrosine kinases, and was originally identified as a component of a fusion protein commonly found in anaplastic large cell lymphomas.^19,20^ Upon ligand binding, ALK activates downstream signaling through several pathways, including RAS/RAF/MEK/ERK, JAK/STAT, PI3K, and PLC, which play a role in regulation of cell proliferation and survival.^19,20^ Fusions between ALK and various partners, commonly EML4 in non-small cell lung cancer and NPM1 in anaplastic large cell lymphoma, lead to constitutive activation of downstream signaling and function as oncogenic drivers.^19,20^ Because of this, ALK inhibitors have been developed to target these fusions and several have obtained FDA approval.^21^ However, while initially successful, ALK inhibitor therapy ultimately fails for many patients, as various resistance mechanisms emerge to circumvent therapeutic efficacy. These resistance mechanisms include ALK missense mutations, as well as copy number alterations, or mutations in alternate pathways that activate bypass signaling.^21–23^

Capturing and visualizing the data related to these complex scenarios is paramount to advancing our understanding of tumor biology and precision oncology. However, data regarding efficacy of targeted therapies are currently primarily captured as they relate to a single alteration, and other tumor-related alterations may be disregarded. As a result, gleaning insight into the relationship between more than one potentially important alterations in a tumor and therapeutic efficacy can prove challenging. To address this, we have incorporated the concept of a “complex molecular profile” into the JAX Clinical Knowledgebase (JAX-CKB), which enables us to relate evidence of therapeutic efficacy to a collection of molecular changes, or molecular profile, which can include single nucleotide variations (SNVs), copy number variations (CNVs), fusions, and/or expression level changes. Thus, the complex molecular profile provides opportunity for a more accurate representation of the genomic conditions influencing therapeutic response as they are reported in the literature, and reduces the potential for erroneous interpretation.

The JAX-CKB is a relational knowledgebase that incorporates integrated data related to cancer-associated genomic variants, therapeutic efficacy, and clinical trials, which is populated largely by manual curation of data from the scientific literature and provides a powerful tool for interpretation of genomic data in cancer.^24^ This differs from a genomic repository or genomic database, such as COSMIC or cBioPortal, that focuses on warehousing genomic data from patient clinical samples, which can be in turn used for large scale analyses of genomic variation in cancer.^25–27^ A knowledgebase, such as the JAX-CKB, can be used as a complement to these genomic repositories, as a platform for interpretation of those variants found in patient samples. The interpretations and in-depth connections provided in the JAX-CKB are primarily sourced from the published literature, and relevant curation methodology is available in the help section on the ckb.jax.org website.

Rather than considering each variant individually, the association of data in the JAX-CKB with complex molecular profiles enables a user to quickly assess the relationship between multiple tumor alterations and their compound impact on therapeutic efficacy. In this way, interpretation of somatic variants can be done on a more holistic level, potentially improving the clinical decision-making process. Here, we present the utility of the JAX-CKB in capturing and analyzing data related to complex molecular profiles, with a specific emphasis on the ability to quickly visualize and analyze data related to therapy resistance.

## Results

### Data relationships in the JAX-CKB

To capture data related to multiple alterations simultaneously, we have designed the JAX-CKB to include a concept we have termed the ‘molecular profile’. Molecular profiles can contain one or more gene variants, including any combination of single nucleotide variants, insertions, deletions, duplications, copy number variants, and/or expression changes. Naming convention for single nucleotide variants, small insertions or deletions, and duplications follows Human Genome Variation Society (HGVS) compliant nomenclature, to reduce ambiguity. The JAX-CKB also incorporates higher order variants to accommodate unspecified variants, such as EGFR mutant, EGFR act mut (for EGFR activating mutation), and EGFR exon 19 deletion. These higher order, or “category” variants enable curation of data where the specific alteration present is not identified.

**Fig. 1:**
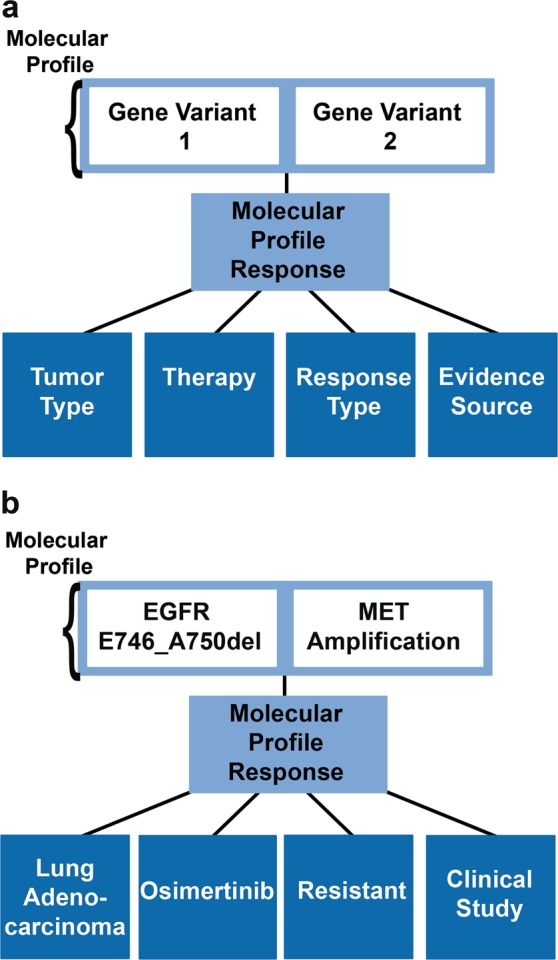
The JAX-CKB complex molecular profile. **a** Structure of the relationship between the complex molecular profile (top) and therapy response, via the molecular profile response. **b** Example of a molecular profile response associated with a complex profile. This complex molecular profile comprises two alterations: EGFR E746_A750del and MET amplification, which was demonstrated in a clinical study (evidence source) to be resistant (response type) to Osimertinib (therapy) in a lung adenocarcinoma (tumor type) patient

Complex molecular profiles can contain variants from multiple genes, such ‘KRAS G12D + PTEN dec exp + TP53 R306*’, where PTEN dec exp refers to decreased expression of PTEN. These molecular profiles are linked to structured free-text efficacy evidence annotations, curated from the published literature, via the ‘molecular profile response’. The molecular profile response comprises the relationship between the molecular profile, a therapy, the relevant indication or tumor type (assigned a disease ontology term and backed by a unique disease ontology ID^28,29^), the evidence source (also termed “approval status” in the JAX-CKB knowledgebase), and response type, incorporating controlled vocabularies for query retrieval (Fig. 1). The evidence source refers to the type of study from which the data was derived, which includes terms related to preclinical and clinical data. The response type refers to the sensitivity or resistance associated with a given molecular profile/therapy/indication combination. We have also incorporated the terms “conflicting” to handle cases where studies exist with conflicting findings, and “unknown” to handle cases where there is no clear response type associated with the curated finding(s). For example, the term “unknown” is used for annotations where therapeutic efficacy results are reported, but the significance as it relates to the associated molecular profile is unclear. Evidence annotations assigned the response type of “unknown” were excluded from analysis.

### Overall data distribution

**Fig. 2:**
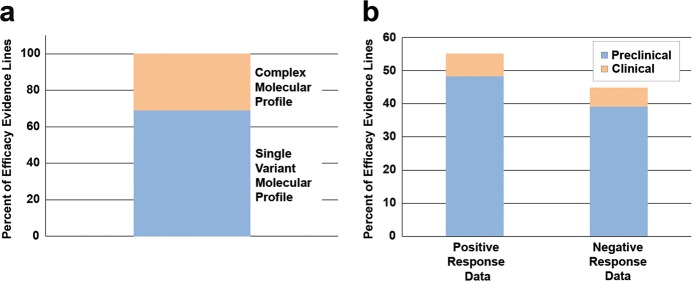
Analysis of efficacy evidence annotations associated with complex molecular profiles in the JAX-CKB. **a** Percent of efficacy evidence associated with complex (orange) or single variant (blue) profiles. **b** Percent of efficacy evidence annotations associated with complex molecular profiles. Percent of efficacy evidence annotations associated with a positive therapeutic response represented by the left bar (55.1%), and annotations associated with a negative response represented by the right bar (44.9%). Data are further divided as they correspond to preclinical (blue) or clinical (orange) data

To gain perspective on the overall landscape of efficacy data curated into the JAX-CKB, we performed analysis on evidence curated to single or complex molecular profiles. Analysis of efficacy evidence in the JAX-CKB demonstrated that 31% of 9271 curated evidence lines are associated with complex molecular profiles (Fig. 2a). Of those, 87.3% of evidence lines are based on preclinical studies (represented by the sum of the blue portions of the bars in Figs. 2b), and 12.7% from clinical studies or clinical trial data (represented by the sum of the orange portions of the bars in Fig. 2b). Of the total efficacy evidence lines associated with complex molecular profiles, 55.1% are associated with a positive response type (those that indicate a positive association between the molecular profile and therapeutic response), and 44.9% are associated with a negative response type (Fig. 2b).

### Evidence related to ALK fusion containing complex profiles

**Fig. 3:**
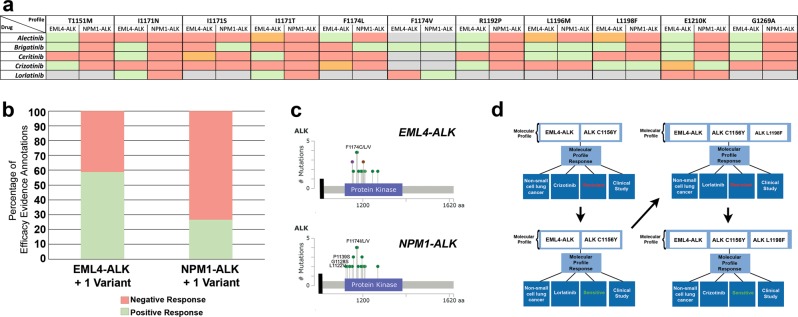
Efficacy evidence in the JAX-CKB corresponding to complex profiles containing ALK fusions. **a** Response of ALK variants (top) within EML4-ALK or NPM1-ALK fusions to ALK inhibitors (left). Green = evidence corresponding to sensitivity, red = evidence corresponding to resistance, orange = conflicting evidence, gray = no available evidence in the JAX-CKB. **b** Percentage of efficacy evidence annotations for complex profiles comprising EML4-ALK plus 1 variant or NPM1-ALK plus 1 variant associated with negative or positive response. **c** Location of variants in EML4-ALK (top) or NPM1-ALK (bottom) associated with negative response efficacy evidence in the JAX-CKB (modified from cBioPortal MutationMapper tool^25,26^). Only the ALK portion of the fusion is displayed. **d** Example demonstrating the structure of complex data from a case study curated in CKB, with varied therapies, response types, and molecular profiles over a treatment course

As a proof of concept on the utility of capturing data related to complex profiles, we examined data in the JAX-CKB that relates to complex molecular profiles comprising ALK fusions in combination with additional ALK variants. Secondary mutations in ALK often occur in tumors with activating ALK fusions following treatment with ALK-targeted therapies and may confer resistance to one or more therapies. Using the JAX-CKB, we analyzed the efficacy data related to response of several ALK mutations to various ALK inhibitors, approved or in clinical development, in the context of EML4-ALK or NPM1-ALK. Analysis of data corresponding to the ALK inhibitor Alectinib demonstrated that several ALK mutations result in a negative response in the context of NPM1-ALK but are sensitive in the context of EML4-ALK (T1151M, F1174L, R1192P, E1210K, G1269A) (Fig. 3a). Efficacy data for EML4-ALK with ALK L1196M, I1171S, or I1171T, or L1198F were conflicting, with data corresponding to both sensitivity and decreased sensitivity to Alectinib. Several mutations also demonstrated differential sensitivity to the ALK inhibitors Brigatinib, Ceritinib, Crizotinib, or Lorlatinib depending on whether they were present in EML4-ALK or NPM1-ALK (Fig. 3a). Complex profiles containing NPM1-ALK plus an additional ALK variant were more likely to be associated with insensitivity to ALK inhibitors, with 73.3% of the curated efficacy evidence lines corresponding to a negative response, whereas 41.1% of EML4-ALK complex profiles were associated with negative response data.

Comparison of the specific ALK complex profiles curated with negative ALK inhibitor response data demonstrated a 57% overlap between variants curated into the JAX-CKB that are associated with negative response data in EML4-ALK and NPM1-ALK, with 19% of variants curated unique to NPM1-ALK, and 24% unique to EML4-ALK (Fig. 3c).

The JAX-CKB also has the capability of capturing data related to more complex scenarios, including data corresponding to therapeutic response in the context of tertiary mutations, occurring in tandem with ALK fusions and secondary ALK mutations. This is exemplified by the representation of data from a clinical case study^30^ as captured in the JAX-CKB (Fig. 3d). In this case study, a non-small cell lung cancer patient harboring EML4-ALK demonstrated an initial response to treatment with Crizotinib, but subsequently demonstrated resistance following the acquisition of the ALK C1156Y mutation (top left). This patient was then treated with Lorlatinib, with a response, but progressed following acquisition of the ALK L1198F mutation (top right). The patient was then treated again with Crizotinib and demonstrated a response (bottom right).

## Discussion

Complex alterations are common in tumors; however, data related to therapeutic efficacy in these contexts are difficult to obtain as the majority of cancer-related efficacy data are represented in the context of a single relevant alteration. Moreover, therapeutic decision-making often focuses, when present, on a single known actionable target alteration, which may relate to poor activity of single-agent targeted therapeutics. Single agent therapies have demonstrated efficacy with some well-known driver alterations, such as Vemurafenib with BRAF V600E mutations in melanoma,^31^ Erlotinib with EGFR exon 19 deletions in non-small cell lung cancer,^32^ or Imatinib with BCR-ABL1 fusions in chronic myeloid leukemia,^33^ leading to FDA approval. However, while initially successful, this efficacy is often short-lived as various resistance mechanisms can occur that activate bypass or parallel pathway signaling, resulting in decreased activity of the targeted therapy.^34–38^ Techniques for non-invasive patient biopsy and detection of mutations present in small cell numbers are constantly improving, allowing physicians to tailor patient treatment accordingly as resistance to targeted therapies develops. However, interpretation of these complex scenarios is non-trivial. Visualization into the various mechanisms potentially impacting therapeutic efficacy, represented as relevant combinations, would provide value for clinical decision making or research purposes. Here, we have demonstrated the utility of capturing therapeutic data related to several relevant alterations, which we have enabled in the JAX-CKB via the concept of a “complex molecular profile”. Efficacy data curated to complex molecular profiles represents a significant and growing area of the JAX-CKB, reflecting the importance of these types of data.

The response to therapy in oncology is impacted by several factors, including the specific combination of alterations present in the tumor. In the case of ALK alterations occurring in the context of an ALK fusion, it is tempting to categorize them as wholly resistant or sensitive to specific ALK-targeted therapies. However, as represented by our analysis, a therapy may be effective in the presence of a given variant when found in EML4-ALK, but not in NPM1-ALK, highlighting the importance of considering the entire profile rather than a single alteration. One limitation to this analysis is the relative impact of the tumor type on the response to a therapy. EML4-ALK is commonly associated with non-small cell lung cancer; NPM1-ALK is most frequent in anaplastic large cell lymphoma. While we did not break down the analysis by tumor type, the majority (86%) of the analyzed data related to preclinical studies using transformed cell lines, expressing either NPM1-ALK-based constructs or EML4-ALK-based constructs, thus effectively normalizing the analysis.

An additional point of consideration is the existence of several distinct EML4-ALK variants, with differential stability and domains and differential response to ALK inhibitors, adding an additional layer of complexity.^39,40^ Shorter EML4-ALK variants have been demonstrated to have higher stability and are less responsive to ALK inhibitors,^40^ which may in part be reflected in the conflicting results seen in studies looking at the sensitivity of different ALK mutations in EML4-ALK to ALK inhibitors. For example, the evidence for sensitivity of I1171S to Ceritinib in CKB comes from a study that expressed this mutation in variant 1 of EML4-ALK,^22^ where the study demonstrating resistance used a construct based on the shorter EML4-ALK variant 3.^41^ Also, while all known EML4-ALK variants include the ALK protein kinase domain as well as the EML4 transactivation domain, the breakpoint and domain composition of EML4 is variable. EML4-ALK variants differ in respect to inclusion of EML4 HELP and/or TAPE domains, which may play a role in response to therapy.^42^ The identity of the ALK fusion partner has also demonstrated role in the sensitivity of ALK inhibitors,^39^ and NPM1-ALK has been shown to be generally less sensitive to ALK inhibition compared to EML4-ALK, supporting the findings reported here.

Of the ALK inhibitors analyzed, five are FDA-approved for use in patients: Alectinib (Alecensa), Crizotinib (Xalkori), Ceritinib (Zykadia), Brigatinib (Alunbrig), and Lorlatinib (Lorbrena); while the others are in various stages of clinical development. Outside of Crizotinib (Xalkori), the ALK inhibitors analyzed are second or third-generation inhibitors, designed to overcome therapeutic resistance. Patients treated with next-generation ALK inhibitors following acquisition of resistant mutations may develop additional tertiary mutations that confer resistance to these newer drugs as well, or potentially resensitize patients to first-generation ALK inhibitors.^30^ The JAX-CKB is designed to capture and display these types of data, potentially aiding in interpretation of complex scenarios. Additionally, the availability of a system such as the JAX-CKB that enables the user to quickly assess mutations that are potentially resistant to targeted therapies, such as specific ALK alterations in the context of an ALK fusion, could potentially aid in clinical trial design and analysis, as patients could be stratified by presence or absence of potentially resistant variants. As data around cancer-related genomic alterations and therapeutic response grows in complexity, the JAX-CKB, through the complex molecular profile, has the capability of structuring those data in meaningful and accurate ways, potentially facilitating more accurate patient-specific therapy decisions.

## Methods

### Database

The JAX-CKB is a relational database that houses integrated knowledge related to genomic variants and targeted therapeutics in oncology. Details related to the overall structure and relationships within the JAX-CKB database have been published previously.^24^ Analysis was performed using version 1.22.2 of the JAX-CKB. The JAX-CKB can be accessed at: https://ckb.jax.org. Curation methodology for data in the JAX-CKB is available in the ‘Help’ section of the website.

### Analysis

Data in the JAX-CKB [accessed on 12/07/2017] was queried to obtain curated efficacy evidence lines, which are free-text annotations summarizing findings from published literature related to therapeutic efficacy, corresponding to single or complex molecular profiles. For analyses of the overall distribution of data in the database, both publicly available and commercially available JAX-CKB content was used for larger sample size. For ALK related efficacy data, the JAX-CKB was queried to retrieve 362 efficacy evidence lines corresponding to complex molecular profiles containing an ALK fusion and additional variants, which was further divided on profiles containing the variant EML4-ALK or NPM1-ALK plus an additional ALK variant for analysis. Data was collated and sorted based on response type, and/or efficacy evidence study source. Data corresponding to positive response type are those molecular profile responses that are assigned the response type of: ‘sensitive’ or ‘sensitive-predicted’; data corresponding to negative response type are those that are assigned the response type of ‘resistant’, ‘resistant-predicted’, ‘decreased response’, or ‘no benefit’. When noted, data was divided by tumor type, with data associated with the tumor type “Advanced Solid Tumor” corresponding to unspecified tumor types or transformed cell lines. Data was also collated by evidence source into preclinical categories (associated with ‘preclinical’, ‘preclinical-cell culture’, ‘preclinical-PDX’, ‘preclinical-cell line xenograft’, ‘preclinical-PDX and cell culture’ terms), or clinical (associated with ‘Phase I’, ‘Phase II’, ‘Phase III’, ‘Clinical Study’, or ‘FDA Approved’ terms). Analysis on data corresponding to the efficacy of specific drugs in the context of EML4-ALK or NPM1-ALK plus a variant were restricted to those variants with curated data for both fusions, and drugs in clinical development or FDA approved.

## Data Availability

Data used for analysis corresponding to ALK related content for this manuscript is available at: https://ckb.jax.org/. The efficacy evidence annotations used for analyses of overall distribution of data for Fig. 2 are from the full JAX-CKB database, which includes both publicly available content (those related to the 86 genes displayed publicly at the time of analysis) as well as data available in the commercial version of the JAX-CKB, which are not available publicly. However, analyses of public content only are consistent with the full JAX-CKB content.
